# *Blastocystis*: A Mysterious Member of the Gut Microbiome

**DOI:** 10.3390/microorganisms12030461

**Published:** 2024-02-24

**Authors:** Mehmet Aykur, Erdoğan Malatyalı, Filiz Demirel, Burçak Cömert-Koçak, Eleni Gentekaki, Anastasios D. Tsaousis, Funda Dogruman-Al

**Affiliations:** 1 Department of Parasitology, Faculty of Medicine, Tokat Gaziosmanpasa University, Tokat 60030, Türkiye; 2 Department of Parasitology, Faculty of Medicine, Aydin Adnan Menderes University, Aydin 09010, Türkiye; erdogan.malatyali@adu.edu.tr; 3 Department of Medical Microbiology, Ankara City Hospital, Health Science University, Ankara 06500, Türkiye; dr.filiz.demirel@gmail.com; 4 Department of Medical Microbiology, Karadeniz Ereğli State Hospital, Zonguldak 67300, Türkiye; cburcakt@yahoo.com; 5 Department of Veterinary Medicine, School of Veterinary Medicine, University of Nicosia, Nicosia 2414, Cyprus; gentekaki.e@unic.ac.cy; 6 Laboratory of Molecular and Evolutionary Parasitology, RAPID Group, School of Biosciences, University of Kent, Canterbury CT2 7NZ, UK; a.tsaousis@kent.ac.uk; 7 Division of Medical Parasitology, Department of Medical Microbiology, Faculty of Medicine, Gazi University, Ankara 06560, Türkiye; alfunda@gazi.edu.tr

**Keywords:** *Blastocystis*, gut microbiome, microbiome modulation, immune modulation, autoimmune disease, gut–brain axis, probiotics

## Abstract

*Blastocystis* is the most common gastrointestinal protist found in humans and animals. Although the clinical significance of *Blastocystis* remains unclear, the organism is increasingly being viewed as a commensal member of the gut microbiome. However, its impact on the microbiome is still being debated. It is unclear whether *Blastocystis* promotes a healthy gut and microbiome directly or whether it is more likely to colonize and persist in a healthy gut environment. In healthy people, *Blastocystis* is frequently associated with increased bacterial diversity and significant differences in the gut microbiome. Based on current knowledge, it is not possible to determine whether differences in the gut microbiome are the cause or result of *Blastocystis* colonization. Although it is possible that some aspects of this eukaryote’s role in the intestinal microbiome remain unknown and that its effects vary, possibly due to subtype and intra-subtype variations and immune modulation, more research is needed to characterize these mechanisms in greater detail. This review covers recent findings on the effects of *Blastocystis* in the gut microbiome and immune modulation, its impact on the microbiome in autoimmune diseases, whether *Blastocystis* has a role like bacteria in the gut–brain axis, and its relationship with probiotics.

## 1. Introduction

*Blastocystis* is one of the most common microbial eukaryotes in the gastrointestinal tracts of humans and animals. Based on small subunit ribosomal RNA (SSUrRNA), the genus is composed of many genetically distinct subtypes (STs) that most likely represent separate species. The current taxonomy of *Blastocystis* is as follows: the kingdom Sar, the phylum Stramenopiles, the class Bigyra, the order Opalinata, the family Blastocystidae and the genus *Blastocystis*; species are not applicable [1]. Stramenopiles comprise over 100,000 species distributed across 21 classes. The majority of described species are diatoms, followed by brown algae, chrysophytes, xanthophytes, and oomycetes. However, unlike most other members of Stramenopiles, *Blastocystis* is neither flagellated nor motile [2]. In this review, the term “*Blastocystis* colonization” is used to define both the natural and experimental infection of hosts. *Blastocystis* can grow abundantly in xenic media and can be easily isolated from fresh fecal samples. However, achieving an axenic culture of *Blastocystis* is a very challenging process [3]. *Blastocystis* is well adapted to the anoxic/microaerophilic gut environment and lacks typical eukaryotic features, including cytochrome-driven mitochondrial electron transport. The organism is usually defined as a strict or obligate anaerobe that encodes genes for oxygen stress, including an alternative oxidase [4] and an SUF mobilization system [5]. Metabolically, *Blastocystis* has a glycolytic pathway whose components are localized in both the cytosol and its mitochondria, and recently, a mitochondrial carrier capable of transporting glycolytic intermediates was discovered, thus bridging the two branches of glycolysis [6].

*Blastocystis* has a global distribution; however, higher frequencies have been reported in developing countries because of poor hygiene, animal handling, or the fecal contamination of food and water [7,8]. The range of genetic diversity in *Blastocystis* is considerably high, and recently, at least 42 STs were identified from various hosts, relying on small subunit ribosomal RNA gene (SSU rRNA) polymorphisms [9,10,11]. In fact, one of the most significant current discussions is the number of STs and the identification of novel subtypes [12]. The genome of *Blastocystis* ST7 was the first to be sequenced in 2011, with data from ST1, ST2, ST4, ST6, ST8, and ST9 becoming available later at various stages of annotation [13,14]. Despite sharing common core genes, some important features, including genome sizes, intron numbers, guanine–cytosine (GC) contents, and gene contents, vary among subtypes [15].

The role of *Blastocystis* in the development of gastrointestinal diseases has also been much disputed despite a considerable number of studies [16,17,18]. *Blastocystis* infection has been associated with non-specific gastrointestinal symptoms such as abdominal pain, diarrhea, nausea, vomiting, bloating, and anorexia, as well as less frequent dermatological complaints like urticaria and severe itching [2,19,20,21]. In vitro studies on *Blastocystis* pathogenesis demonstrated that it can attach to the intestinal mucosa, increase intestinal permeability by secreting cysteine proteases, degrade secretory immunoglobulin A (IgA), induce the secretion of inflammatory cytokines such as interleukin-8, and cause the apoptosis of host cells [1,22]. In general, the prevalence of *Blastocystis* has been reported to be higher in healthy populations compared to individuals with ulcerative colitis (UC) or irritable bowel syndrome (IBS) [23,24]. *Blastocystis* resides in the human intestine for a long period of time without causing any symptoms, encouraging the question of whether it should be considered a pathogen or a commensal microorganism [25,26]. Nonetheless, the eradication of *Blastocystis* is considered necessary in cases where it is the sole protist agent and the patient’s complaints persist [27]. There are limited studies revealing information regarding the actual abundance of *Blastocystis* in the host. In a study by Poirier et al., the parasite density of *Blastocystis*-positive samples was evaluated using a qPCR. *Blastocystis* numbers in hosts were reported to vary between <10^2^ and >10^7^ *Blastocystis*/g fecal sample [28].

The gut microbiome refers to the collection of bacteria, viruses, archaea, and eukaryotes that colonize the gastrointestinal tract, primarily the large intestine. This highly dynamic and complex ecosystem plays a crucial role in maintaining human health and has various physiological functions. It is currently accepted that the human gut microbiome is first acquired and established before or during birth, with the mode of delivery, ethnicity, and host genetics playing roles in its composition [29,30]. In addition, various external factors such as diet, nutritional status, prenatal events, geographical location, antibiotic treatment, and age contribute to establishing the gut microbiome throughout human life [31,32,33,34,35]. The microbiome reaches a “balanced” state with high taxonomic microbial diversity and richness in the following years of life, forming a commensal relationship with the host [36]. The Human Microbiome Project (HMP) and the Metagenomics of the Human Intestinal Tract (MetaHIT) project, as well as the development of novel technologies such as 16S rRNA gene metabarcoding, have improved our understanding [37,38]. The study of the gut microbiome has become a major area of interest in various disciplines. These days, some define the microbiome as a novel multicellular “organ” which interacts closely with its host [39]. The gut microbiome has numerous important functions including digestion, nutrient production, immune system regulation, gut barrier function for pathogens, and the regulation of metabolic activities; therefore, maintaining a healthy and diverse gut microbiome is essential for overall well-being [40,41]. The term “dysbiosis” can be defined as a persistent imbalance in the gut microbial community and can lead to various chronic conditions. Integrative analyses of the gut microbiome in humans and laboratory animals have offered possible relationships with many chronic diseases such as autoimmune disorders, obesity, diabetes, IBS, metabolic syndrome, depression, and allergy [42,43,44,45,46,47].

Single-celled eukaryotes constitute an important and heterogeneous group within the human intestinal microbiota. A major discussion point revolves around the categorization of these species as pathogenic, commensal, beneficial, or opportunistic pathogens. The well-known gut-related protozoa in humans are *Blastocystis*, *Dientamoeba fragilis*, *Giardia intestinalis*, *Entamoeba histolytica*, and *Cryptosporidium* spp. Among these, the last three significantly contribute to acute gastroenteritis and diarrheal diseases on a global scale [48]. However, many intestinal protist species, such as *Endolimax nana*, *Entamoeba polecki*, *Iodamoeba butschlii*, and *Chilomastix mesnili*, are non-pathogenic and might even be beneficial inhabitants of the gut [49]. Presently, at least eight species of *Entamoeba* spp. (*E. polecki*, *E. gingivalis*, *E. chattoni*, *E. histolytica*, *E. dispar*, *E. hartmanni*, *E. moshkovskii*, and *E. Bangladeshi*) have been identified in human samples, while *E. histolytica* is the only species with well-established pathogenicity [50]. A metagenomic approach that included samples from different countries revealed higher frequencies of *Entamoeba* spp., *Blastocystis*, and some other protozoan genera in healthy individuals [51]. Most retrospective studies have reported conflicting results regarding the roles of *D. fragilis* and *Blastocystis* in the development of gastrointestinal diseases [52,53]. Recent investigations on the microbiota have provided novel approaches to understanding the pathogenicity of intestinal protozoa.

There is a growing body of literature that emphasizes the importance of *Blastocystis* in the human gut microbiome [54,55,56,57]. Metagenomic studies have shown an association with increased abundances of the phylum Bacillota (syn. Firmicutes) and the class Clostridiales in the gut microbiomes of *Blastocystis*-colonized individuals, as well as a decreased abundance of *Bacteroides* [58,59]. However, *Blastocystis* infection has been linked to gut microbiome imbalance in certain gastrointestinal diseases such as IBS–constipation and inflammatory bowel disease (IBD) [60,61,62]. In addition, few studies have investigated *Blastocystis* subtype and microbiome interactions [55,57]. In general, *Blastocystis* is a common eukaryote in the intestinal microbiome of healthy humans. Its presence is linked with the high diversity and richness of bacterial communities [57]. However, a systematic understanding of how *Blastocystis* affects the gut microbiome and vice versa is still lacking. The main subjects addressed in this review are *Blastocystis* and gut microbiome modulation, immune modulation, autoimmune diseases, and, finally, the gut–brain axis.

## 2. *Blastocystis* and the Gut Microbiome

### 2.1. The Effect of Blastocystis on Gut Microbiome Modulation

*Blastocystis* colonization is thought to be related to changes in the gut bacterial microbiome [63]. Recent studies indicate that *Blastocystis* infection may be associated with alterations in the abundances of both beneficial and harmful intestinal bacteria. Research on the relationship between asymptomatic *Blastocystis* infection and intestinal bacterial composition is ongoing, although this association still needs to be fully understood [57,64,65]. Behboud et al. have reported that the mean relative abundances of *Bifidobacterium* and *Lactobacillus/Enterococcus* (beneficial bacteria) groups and *Peptostreptococcus productus* and *Escherichia coli* (harmful bacteria) were upregulated significantly, while the relative abundances of *Bacteroides fragilis* (*B. fragilis*) and *Enterococcus* sp. were downregulated considerably in those with *Blastocystis* compared to a control group [64]. According to a study by Di Cristanziano et al., in patients with *Blastocystis*, there was a consistent presence of bacterial genera linked to healthy status, including *Eubacterium rectale* and *Eubacterium coprostanoligenes* groups, as well as *Roseburia* and *Succinivibrio*. Nevertheless, their relative abundances were consistently lower compared to the control group [66].

**Figure 1:**
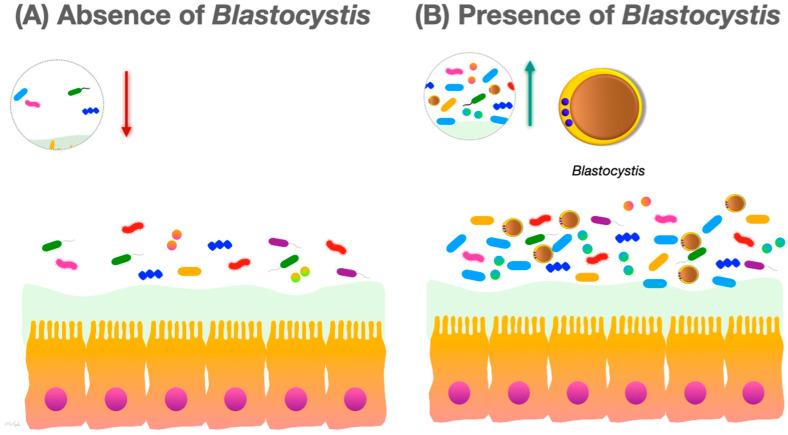
(**A**) In the absence of *Blastocystis*, the gut microbiota species richness and community evenness are lower; (**B**) in the presence of *Blastocystis*, the species richness and community evenness of the gut microbiota increases.

Many studies report that colonization with *Blastocystis* is associated with increased diversity of the human intestinal bacterial microbiota (Figure 1). For instance, Audebert et al. reported a higher abundance of Clostridia and a lower abundance of Enterobacteriaceae in the fecal microbiota of patients colonized with *Blastocystis*, concluding that *Blastocystis* colonization is generally associated with healthy intestinal microbiota [67]. In a study aiming to evaluate the effect of *Blastocystis* on gut microbiota in healthy children, the diversity of intestinal microbiota and the proportion of beneficial bacteria were found to be higher in children colonized with *Blastocystis* than in children not colonized with the organism [68]. These results follow those of Alzate et al., who also found that *Blastocystis* was associated with a significant increase in bacterial richness in children [69].

There are limited data in the literature on the relationships between *Blastocystis* STs and the gut microbiota. *Blastocystis* ST1 is one of the most commonly found STs in humans worldwide [70,71,72]. Some studies have demonstrated that ST1 has beneficial effects on the host gut microbiome and immune system. Deng et al. showed that colonization with *Blastocystis* ST1 could increase the levels of *Alloprevotella* and *Akkermansia*, which are beneficial bacteria for gut health, in a murine model [73]. *Blastocystis* ST3, another very common subtype, has been shown to cause an increase in beneficial bacteria such as Bacillota (syn. Firmicutes) and Bacteroidota (syn. Bacteroidetes) in the host gut microbiome, and it has been reported that this may indirectly be beneficial to the host immune response [74]. In a study examining the effect of *Blastocystis* ST3 colonization in a rat model, no significant influence on bacterial alpha diversity was observed before inducing colitis. However, after colitis induction, higher bacterial diversity was observed in rats with long-term *Blastocystis* ST3 colonization [63]. Recently, it was also reported that *Blastocystis* ST4 is beneficial for the gut as it increases the diversity of the gut microbiome [75,76]. *Blastocystis* ST4 has been found to promote the abundance of groups of bacteria belonging to *Akkermansia* spp., the family Lachnospiraceae, and the class Clostridia, all of which are considered beneficial to gut health, and to also inhibit the proliferation of *Bacteroides* spp., *Escherichia* spp., and *Shigella* spp. in the intestine, resulting in the alleviation of intestinal inflammation [75,76]. These results reflect those of Deng et al., who also found that colonization with *Blastocystis* ST4 may modify the intestinal microbiome and increase the accumulation of Th2 and Treg cells in the intestinal mucosa in a mouse model of induced colitis [75]. It has also been demonstrated that while *Blastocystis* ST4 colonization increases beneficial bacteria, it inhibits the proliferation of *Bacteroides vulgatus*, which is pathogenic for the intestine, when co-incubated with intestinal bacteria [77]. It has been observed that healthy individuals colonized with *Blastocystis* ST4 have high abundances of bacterial genera such as *Sporolactobacillus* and *Candidatus* Carsonella in their gut microbiome, while a reverse correlation was observed with *Akkermansia* [78,79]. Although ST7 is less common in humans worldwide than other STs of *Blastocystis*, an ST7 isolate has been reported to have pathogenic properties in in vitro and in vivo studies [62]. In addition, it has been reported that *Blastocystis* ST7 can disrupt the microbiotic balance in the gut microbiome population, especially by reducing *Bifidobacteria longum* (*B. longum*) and *Lactobacillus brevis* (*L. brevis*) [62]. Both *L. brevis* and *B. longum* have been found to benefit the gut microbiomes of IBS and IBD patients in addition to other beneficial gut microbiome species [80,81,82]. Even et al. verified that the colonization of *Blastocystis* has a major impact on the higher-level taxonomic diversity of the gut microbiota. The authors also found that the relative abundances of Ruminococcaceae and Clostridiales were higher in patients colonized with *Blastocystis*. Interestingly, it was shown that patients with multiple STs had a higher diversity of gut bacteria than those with just one ST [56].

It is considered that the composition of the gut microbiome plays a crucial role in the pathogenesis of certain diseases such as IBD, which is a chronic inflammation of the gastrointestinal tract. Although some studies have reported a high prevalence of *Blastocystis* in patients with IBD, the relationship between the organism and the disease is still controversial [83]. Deng et al. revealed that *Blastocystis* ST7 was associated with a decrease in beneficial bacteria such as *Bifidobacterium* and *Lactobacillus* and could lead to an increase in the severity of colitis in their murine model. They also reported that *Blastocystis* ST4 can decrease the severity of colitis by modulating the gut microbiome [83].

In contrast, Nagel et al. reported no association between *Blastocystis* and the gut microbiome in diarrhea-predominant IBS patients [84]. In another study focusing on cirrhotic patients with hepatic encephalopathy (HE), it was proposed that an inverse association existed between *Blastocystis* and HE severity. The authors reported that the alteration in bacterial diversity and the presence of *Blastocystis* may be significant factors in the pathophysiology of HE, highlighting a need for more research on this subject [85].

### 2.2. The Effect of Blastocystis on Immune Modulation

The gut microbiome plays an essential role in the health and disease status of the host. It is now known that it contributes significantly to the pathogenesis of autoimmune diseases, with the deterioration of the gut microbiome being linked to the dysregulation of the immune system [86]. The pathogenic potential of *Blastocystis*, its clinical significance, and its potential effects on the host immune system are still debated [57]. Whether *Blastocystis* is pathogenic or non-pathogenic depends on factors such as its interaction with the human gut microbiome, the subtype, and the human immune response regulators or modulators involved [75]. While *Blastocystis* colonizes the human gut and does not cause any infection, this situation can change in the event of a disruption to the immune system or gut microbiome balance [87]. An investigation of the metabolic profiles of *Blastocystis* carriers and non-carriers revealed differential levels of certain amino acids (Ala, Gly, His, Ile, Met, Thr, Try, and Val) in fecal samples collected from individuals from different countries. These amino acids are considered inflammatory biomarkers if their abundance in the gut is increased. The decrease in these amino acids in *Blastocystis*-positive individuals may indicate that the organism assumes an anti-inflammatory role in the intestine [88].

**Figure 2:**
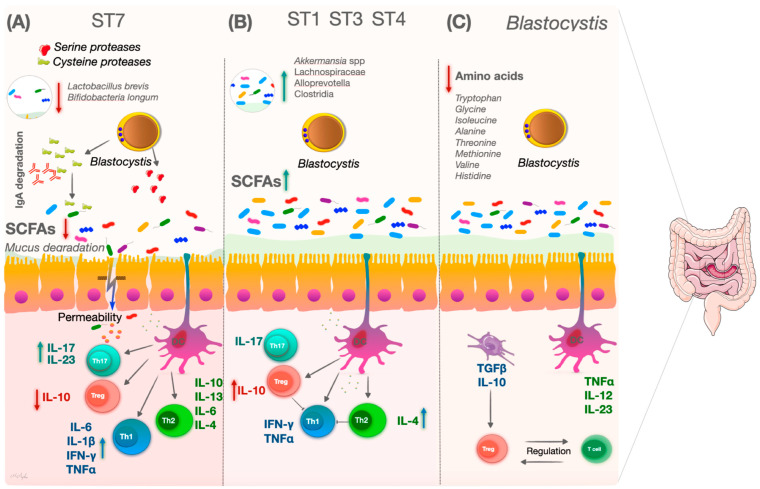
A general graphical overview of the impact of the gut microbiome on the immune response in *Blastocystis* infections and some of its subtypes. (**A**) The gut microbiome associated with *Blastocystis* ST7 can establish a pro-inflammatory environment by interacting with epithelial and dendritic cells (DC). (**B**) *Blastocystis* ST1, ST3, and ST4 increase the diversity of the gut microbiome and promote an anti-inflammatory state in the intestinal mucosa. (**C**) The decrease in some amino acids in the gut microbiome in the presence of *Blastocystis* may provide a balance in immune modulation.

One of the areas of interest in *Blastocystis* research, along with recent work, is the insight into its potential to modulate the host immune system. Research suggests that *Blastocystis* infection can elicit various immune responses, including both pro-inflammatory and anti-inflammatory responses. However, the exact nature and significance of these immune responses are not fully understood [89]. Recent studies have revealed how some *Blastocystis* subtypes modulate the gut microbiome composition and how this change in the gut microbiome affects the immune response (Figure 2) [87,90,91].

The gut microbial ecosystem is crucial for the modulation and regulation of the immune system [92,93]. Mucin, a thick and sticky glycoprotein, is produced by goblet cells that secrete mucus in the body, especially in the gastrointestinal tract. Cysteine proteases, produced by pathogenic parasites, cause the breakdown of mucin, creating gaps between colon epithelial cells and thus making the invasion of the underlying host tissue possible [94]. The gut microbiome can support the development of T regulatory cells (Tregs) by producing short-chain fatty acids (SCFAs) and regulating Th2 immune responses during parasite infection. More specifically, *Blastocystis* ST4 has been associated with increased abundances of bacteria such as *Akkermansia* spp. and SCFA-producing bacteria associated with increased SCFA production, which can provide energy to goblet cells [49,95]. These data indicate subtype-specific effects of *Blastocystis* on immune modulation. Moreover, regarding host–microbiome interactions, the increase in diverse bacteria in the gut microbiota of *Blastocystis* ST1 and ST4 carriers causes SCFA production, which is important for the immune system overall and its modulation [73,96].

Immunoglobulin A is crucial in the mucosal defense of the gastrointestinal tract as it provides immune protection against microbial pathogens [97]. The release of cysteine protease by *Blastocystis* ST7 and aspartic proteases by ST4 has been shown to mediate the degradation of IgA and subsequently modulate the host immune response [87,98]. It has been demonstrated that *Blastocystis* ST4 cysteine proteases induce the upstream synthesis of interleukin (IL)-8 through the nuclear factor-κB (NF-κB) pathway [99]. An increase in the proinflammatory chemokine IL-8 and granulocyte–macrophage colony-stimulating factor (GM-CSF) in human colon carcinoma cells with a *Blastocystis* ST1 co-culture has been reported [2]. In another study involving colonic epithelial cells, *Blastocystis* ST1 modulated the immune system by stimulating IL-8 release [100]. In the presence of colonization by *Blastocystis* ST7, a cascade of mitogen-activated protein kinases (MAPKs), an important signaling pathway in innate immunity, induced the upregulation of the pro-inflammatory cytokines IL-6, IL-1β, and tumor necrosis factor-α (TNFα) in murine macrophages [57,101]. These cytokines play key roles in initiating and regulating the immune response. Additionally, *Blastocystis* has been found to activate immune cells, such as macrophages and dendritic cells, which are involved in the innate immune response [89].

Numerous studies in recent years have shown that Th1 and Th2 cells play distinct roles in the mediation of immunological responses [102]. Interferon-gamma (IFN-γ), IL-12, IL-2, and TNF-α are primarily secreted by Th1 cells, which additionally regulate cellular immunity. Th2 cells are essential for Th1 differentiation and the Th1 response. Th2 cells play a key role in mediating humoral immunity by primarily producing IL-4, IL-10, IL-13, and IL-6 [103]. Colonization with *Blastocystis* ST1, ST3, and ST4, the most common subtypes of *Blastocystis*, was associated with enhanced potent Th2 and Treg immune responses in a murine model of experimental colitis. Colonization with ST1, ST3, and ST4, has been reported in studies to have a beneficial effect on host health by regulating the gut microbiome composition and adaptive immune responses [63,73,75].

Deng et al. indicated that ST1 colonization could induce Th2 and Treg cell responses in normal, healthy mice [73]. Long-term *Blastocystis* ST3 colonization was reported to modify the appearance of induced colitis in a rat model of intestinal inflammation, whereas short-term colonization had no effect. In addition, it has been suggested that prolonged colonization with *Blastocystis* ST3 may be protective against intestinal inflammation by promoting faster recovery due to a significant decrease in inflammatory markers such as TNFα and IL-1β [63]. Moreover, *Blastocystis* ST4 induces Th2 immune responses and increases the production of IL-4, IL-5, IL-13, and IL-10, thereby causing the suppression of inflammatory responses in colonic mucosal tissues [76].

The overall immune modulation effects of *Blastocystis* are complex and can vary depending on the specific subtype of the parasite, the host immune status, and other factors. When *Blastocystis*-positive and *Blastocystis*-negative patients with chronic urticaria were compared, patients infected with ST3 and ST2 had a higher abundance of IgE. However, there was no relationship between the *Blastocystis* STs of healthy individuals and patients with chronic urticaria [21]. Furthermore, the clinical implications of these immune responses are still not well understood.

It is worth noting that *Blastocystis* is often found in healthy individuals without any symptoms, suggesting that it may have a commensal symbiotic relationship with the host in certain cases [63,74,104]. However, more research is needed for a better understanding of the immunomodulatory effects of *Blastocystis* and their clinical significance in different individuals.

### 2.3. The Interaction of Blastocystis and the Gut Microbiome in Autoimmune Diseases

Autoimmune diseases (ADs) occur when cells of the immune system attack the host’s cells and tissues, resulting in chronic inflammation. In the last decade, it has become known that environmental factors trigger ADs in genetically predisposed individuals [105]. The gut microbiome, which consists of trillions of microorganisms inhabiting the gastrointestinal tract, plays a critical role in regulating the immune system and maintaining gut health. It has been suggested that imbalances, either an increase or decrease in the specific taxa of the gut microbiome, may contribute to the development of ADs [106]. A disturbed balance in the gut microbiome may be associated not only with intestinal ADs (IBD, IBS, celiac disease, and autoimmune gastritis, etc.) but also with extra-intestinal ADs (multiple sclerosis, rheumatoid arthritis (RA), type 1 diabetes, and systemic lupus erythematosus (SLE)).

Few studies have investigated the relationship between ADs and the presence of *Blastocystis*. These include ADs such as SLE, RA, spondyloarthritis (SpA), IBD, UC, Crohn’s disease (CD), and IBS [107,108,109,110]. However, the richness and diversity of the gut microbiome in association with *Blastocystis* and ADs have only been examined in SpA and IBS patients [111].

Spondyloarthritis comprises a group of rheumatic diseases with differential clinical features, such as ankylosing spondylitis (AS), reactive arthritis (ReA), and psoriatic arthritis (PsA), along with inflammatory bowel disease-associated SpA, uveitis, and dermatological and gastroenterological involvement [112]. A prevalent feature in many inflammatory diseases, including SpA, is gut microbial dysbiosis. Patients with SpA showed a decreased fecal abundance of *Faecalibacterium prausnitzii* and an increase in *B. fragilis* [113]. Regarding the gut microbiome composition of SpA patients, the main results from a meta-analysis showed increased frequencies of Bacteroidaceae and Enterobacteriaceae in the phylum Pseudomonadota (syn Proteobacteria), while the gut microbiome diversity in the phylum Bacteroidota (syn Bacteroidetes) showed decreases in Bacteroidales and *Akkermansia* [114]. There is a growing number of studies on the link between the gut microbiome and SpA, and several research investigations have demonstrated that the microbial profiles of SpA patients and healthy people differ [115,116,117]. *Blastocystis*-positive SpA patients showed significant increases in Pseudomonadota (syn Proteobacteria), the class Gammaproteobacteria, the family Succinivibrionaceae, and the genus *Succinivibrio*. However, in *Blastocystis*-negative SpA patients, there were significant increases in the *Bacilli* class, the order Lactobacillales, the Lactobacillaceae and Clostridiaceae families, and the genera *Lactobacillus* and *Clostridium* [111]. While *Blastocystis*-positive healthy individuals showed an increased diversity of the gut microbiome, no such increase was noted in the intestinal diversity of SpA patients [111]. These findings highlight the importance of *Blastocystis* as a typical component of a balanced gut microbiome [25,79].

Irritable bowel syndrome is a common functional gastrointestinal disorder characterized by abdominal pain, discomfort during defecation, and changes in the gut microbiome [118]. Some studies have reported that the gut microbiome of IBS patients had a significantly increased number of bacteria in the families Enterobacteriaceae and Bacteroides compared to healthy controls. Moreover, a significant increase in the family Lactobacillaceae in IBS patients has been reported [119,120]. A review of the relationship between IBS and the gut microbiome revealed that the genera *Faecalibacterium* and *Bifidobacterium* were significantly reduced in IBS patients [121]. A meta-analysis of 13 publications confirmed the lower abundance of *Bifidobacterium* in IBS patients, along with decreased *Lactobacillus* and *F. prausnitzii* [122]. In other studies, the proportion of *Bifidobacterium* in intestinal microbiota decreased in *Blastocystis*-positive individuals with IBS, while a decrease in *F. prausnitzii* in healthy *Blastocystis*-positive individuals was noted [60,123].

Recently, gut microbiome studies have shown greater abundances and higher gut richness of the Clostridia class, the families Ruminococcaceae and Prevotellaceae, and the *Faecalibacterium* and *Roseburia* genera in individual patients colonized with *Blastocystis* [57,67]. However, individuals not colonized with *Blastocystis* exhibited a higher abundance of Bacteroides [74]. Additionally, the increase in Bacteroides in the gut microbiome in people with various diseases, such as celiac disease and colorectal cancer, appears to be associated with low bacterial diversity [124,125,126,127]. These studies indicate that individuals colonized with *Blastocystis* have been associated with a richer and more diverse gut microbiome.

The interaction between *Blastocystis*, the gut microbiome, and ADs is a complex and evolving area of research. While some studies suggest possible connections, the mechanisms and clinical significance of these interactions remain unclear. Further research is needed to better understand the role of *Blastocystis* in ADs and its potential impact on gut health and the immune system. In individuals with ADs, the presence or absence of *Blastocystis* may cause changes in the balance of the gut microbiome. In addition, the different subtypes of *Blastocystis* should be considered along with the many factors that contribute to developing ADs.

## 3. *Blastocystis* and the Gut–Brain Axis

The communication between the brain and the gut microbiome is bidirectional and is termed the “gut microbiome–brain axis”. Communication along the gut–brain axis is mediated by various transmission systems, including the enteric nervous system, central nervous system, immune system, and endocrine system [128]. Maintaining a good balance between the gut microbiome and the brain is important for the host [80]. Various biochemical and metabolic processes must occur in order to maintain the gut–brain axis balance [129]. During these processes, signals in the gut microbiome modulate aspects of homeostasis through pathways of communication between the gut and the brain via the vagus nerve, metabolites such as SCFAs, the endocrine system, the immune system, and neurotransmitters such as serotonin, dopamine, acetylcholine, glutamate, γ-aminobutyric acid (GABA), and noradrenaline [130].

Although research has been conducted on the relationship between parasite manipulations and insect parasite interactions with the central nervous system (CNS), there have not been many studies on the interaction of the vertebrate host CNS and parasites [131,132]. The understanding of the interaction of the host CNS and parasites has increased recently with the development of the new and developing field of neuro-parasitology. Parasites can significantly affect the functioning of the host organism, including the immune response and the gut–brain axis, resulting in altered host behavior [133]. *Echinococcus granulosus*-derived ESPs (excretory–secretory products) affect cognitive function and the gut microbiome–brain axis as they have been demonstrated to alleviate dysbiosis and ameliorate cognitive decline in obese mice [134]. Another study revealed that *Hymenolepis diminuta* positively affected the spatial memory and new object recognition of the infected animal [131].

Despite the uncertainty surrounding the parasitic nature of *Blastocystis*, studies such as the above can shed light on the gut–brain axis relation to *Blastocystis* colonization/infection. There have been a limited number of studies showing the mechanisms through which the presence of *Blastocystis* in the intestine might influence the cognitive behavior of the host. In a study conducted by Defaye et al., a possible relationship between *Blastocystis* infection, colonic hypersensitivity, behavioral disorders, and gut microbiota changes using a rat model was investigated. In the study, animals infected with *Blastocystis* were associated with colonic hyperresponsiveness, anxiety, and depressive-like behavior [135]. In another study, the transplantation of human *Blastocystis* strains into mice resulted in changes in cognitive function and prefrontal cortex gene expression [136]. The relationship between *Blastocystis* ST1-4 and 7, the Bacillota/Bacteroidota (syn Firmicutes/Bacteroidetes) ratio (F/B ratio) of the fecal microbiota, and chronic stress was investigated in a population of Mexican university students. It was observed that colonization with ST4 was associated with a protective role in chronic stress [55]. Individuals colonized with *Blastocystis* ST4 have been associated with a richer and more diverse gut microbiome [79]. The relationship between *Blastocystis* subtypes, chronic stress, and IBS may need to be balanced by the CNS and gut microbiome [137]. A recent study suggests that *Blastocystis* may have the ability to influence the host’s behavior and mood through the tryptophan synthesis pathway [138].

**Figure 3:**
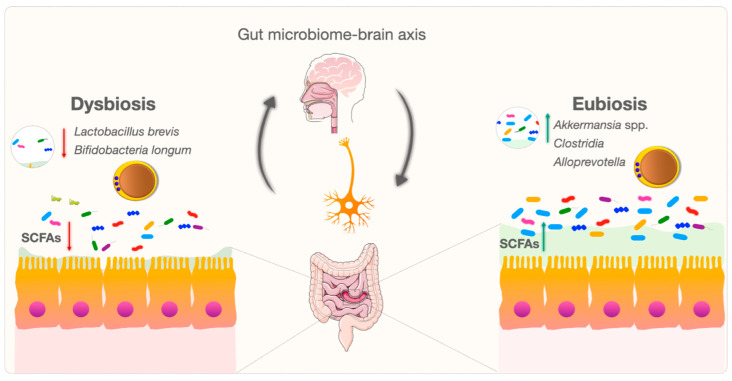
Bidirectional modulation and interaction of the gut microbiome–brain axis between *Blastocystis* and the gut microbiome.

The gastrointestinal system is a complex and dynamic environment. *Blastocystis* exhibits broad genetic diversity, and the mechanisms and relationships between various subspecies and eubiosis/dysbiosis are being investigated [138,139,140,141]. Changes in gut microbiota species and critical metabolite levels in *Blastocystis*-colonized individuals may produce various potent signaling molecules in tryptophan metabolism [138,142]. These molecules may influence the gut microbiome–brain axis by altering tryptophan levels in gastrointestinal and neurological signaling pathways [138,143]. *Blastocystis* may also contribute to the balance of the bidirectional gut–brain axis (Figure 3). *Blastocystis* needs to be further considered as a new and mysterious actor in gut microbiome–brain axis research.

## 4. *Blastocystis* and Probiotics

Probiotics are specific microorganisms that have beneficial effects on health. The most commonly used probiotics are specific strains from lactic acid bacterial species, especially *Lactobacillus* strains (*Streptococcus thermophilus*, *Lactococcus lactis*, *Enterococcus faecium*, and others) and *Bifidobacterium* strains and the yeast *Saccharomyces boulardii* (*S. boulardii*). Probiotics can modulate the microbiota and immune response of the host and inhibit the proliferation of parasites, leading to reduced parasitological loads and clinical improvement. Moreover, probiotics can increase the abundance of beneficial bacteria in the microbiota, change the environmental conditions to become less favorable for pathogens, compete with pathogens for nutrients and adhesion sites pathogens, negatively affect pathogens with their useful secretions (i.e., bacteriocins, lactic acid, hydrogen peroxide, etc.), inhibit bacterial toxins, increase mucus secretion, and induce mucosal immunity [144,145,146,147,148,149,150,151]. Although the relationship between probiotics and parasites has been investigated in various studies [144,148,152,153,154,155,156,157,158], there are very few reports related to *Blastocystis* and probiotics.

*Blastocystis* infections can occur in different forms ranging from asymptomatic to severe. Furthermore, the detection of *Blastocystis* in a stool sample does not necessarily mean that treatment is required. Its presence can be associated with infection or colonization whereby *Blastocystis* is a member of the healthy gut microbiome. This variability in outcomes could be due to different subtypes, the immunological response of the host, and gut microbial diversity [25,58,62,63,67,73,77,159]. If treatment is decided upon in the required symptomatic group (gastrointestinal symptoms; dermatological disorders involving acute/chronic urticaria and itching), the first choice is metronidazole. However, in some cases, failure to respond to metronidazole, the development of resistance, reinfection, or drug-related side effects make treatment difficult. Different antibiotics and combinations can be recommended for treatment (trimethoprim/sulfamethoxazole, paromomycin, secnidazole, tinidazole, and ornidazole), but in vitro sensitivity studies are very few, and studies are limited [19,27,160,161,162,163,164]. At this stage, probiotics may be recommended as a sole treatment option or as a support for treatment.

Dinleyici et al. compared therapies with *S. boulardii* and metronidazole in symptomatic children with a *Blastocystis* infection. They assessed clinical and parasitological cures in both study groups. While both metronidazole and *S. boulardii* demonstrated potential beneficial effects in treating *Blastocystis* infection, no statistically significant difference was found between the two treatment groups [165].

Angelici et al. documented a case report of a symptomatic *Blastocystis* infection caused by contaminated water. The patient had an intolerance to nitroimidazole derivatives, so metronidazole could not be used as the treating agent. Initially, a probiotic containing *Lactobacillus* and *Bifidobacterium* was used, but it did not solve the patient’s problems. The administration of a different probiotic containing *S. boulardii* resulted in successful treatment [166].

Méabed et al. investigated the therapeutic effect of *S. boulardii* in experimental rats which were infected with the most common subtype of *Blastocystis* (ST3). The authors compared the results of parasitologic reduction, histopathological status, and the level of mRNA expression for the proinflammatory cytokines IL-6, IL-8, TNF-α, and inducible nitric oxide synthase (iNOS) on different therapy groups (*S. boulardii* (live), *S boulardii* (extract), metronidazole, co-therapy (metronidazole + *S. boulardii*), and a placebo). The co-therapy involving metronidazole and *S. boulardii* demonstrated a more favorable effect compared to the other treatments. The live *S. boulardii* had a significant beneficial effect on the local immune response of the colonic mucosa, such as goblet cell hyperplasia, as well as lower levels of proinflammatory cytokines and iNOS [167].

Lepczyńska et al. reported that the lactic acid-producing probiotic bacteria *Lactobacillus rhamnosus* and *Lactococcus lactis* significantly inhibited the growth of *Blastocystis* ST3 on xenic and axenic cultures. In the same study, they also investigated the relationship of *Enterococcus faecium* (which can also be used as a probiotic agent), *E. coli*, *Candida albicans*, and *Candida glabrata* with *Blastocystis* in vitro. Lactic acid-producing bacteria began inhibiting the growth of *Blastocystis* on the second day of the study. In contrast, co-culture with *E. coli* and *E. faecium* initially increased *Blastocystis* in the first two days but started to impede its growth after three days. By the fifth day, both *E. coli* and *E. faecium* demonstrated inhibitory effects on *Blastocystis* growth. The presence of *Candida* species had a limited and statistically insignificant effect on the growth of *Blastocystis*. However, it was indicated that *L. rhamnosus* and *L. lactis* may have the potential to be used as probiotics in *Blastocystis* prophylaxis or as a support for treatment [168].

The possible effects of probiotics on *Blastocystis* along with the type and dose of probiotic used for treatment remain unclear. In addition to the views that probiotics have treatment potential for *Blastocystis*, some studies argue that *Blastocystis* is a member of the healthy microbiota and that some *Blastocystis* subtypes may themselves be used as probiotics in the future [159]. This may also be an intriguing research subject. In the future, more successful results can be achieved with the use of probiotics designed by performing personalized microbiome analyses. Additional extensive studies are needed to achieve a comprehensive understanding.

## 5. Conclusions and Perspectives

*Blastocystis* is an important component and potential modulator of the human gut microbiome. This organism modulates the abundance of certain bacterial species and the Bacillota/Bacteroidota ratio. This overall review of recent data provides further support for the hypothesis that *Blastocystis* is a commensal eukaryote and may be an indicator of a healthy and balanced microbiome. However, these results may be somewhat limited by the study population and the methodology used to analyze the microbiome. Another finding that stands out from the earlier findings is that *Blastocystis* might have a subtype-dependent effect on the microbiota. An interesting focus in recent *Blastocystis* research is its potential to modulate the immune system. *Blastocystis* can trigger both pro- and anti-inflammatory cytokines in the host. On the other hand, *Blastocystis* may have immunomodulatory effects that could dampen the immune response. The metabolites and microbial changes could, in turn, potentially affect the production of neurotransmitters and other signaling molecules, thereby influencing brain function and behavior. While research on the relationship between asymptomatic/symptomatic *Blastocystis* infection and intestinal bacterial composition is ongoing, it still needs to be fully understood. However, there is an indication that *Blastocystis* infection may be associated with alterations in both beneficial and harmful intestinal bacteria. Further research on *Blastocystis* and the microbiome holds great promise for unravelling the complex host–protist interactions, understanding their clinical significance, and developing novel therapeutic agents such as probiotics.

## Data Availability

Not applicable.
